# Similar results after five years with the use of the Fitmore or the CLS femoral components: a randomized study of 35 patients

**DOI:** 10.1302/2633-1462.45.BJO-2023-0007.R1

**Published:** 2023-05-03

**Authors:** Karin Rilby, Maziar Mohaddes, Johan Kärrholm

**Affiliations:** 1 Department of Orthopaedics, Institute of Clinical Sciences, Sahlgrenska Academy, University of Gothenburg, Mölndal, Sweden; 2 Department of Orthopaedic Surgery, Sahlgrenska University Hospital, Gothenburg, Sweden; 3 Swedish Hip Arthroplasty Register, Gothenburg, Sweden

**Keywords:** Total hip arthroplasty, Short stems, Radiostereometric analysis, femoral components, hips, patient-reported outcome measures (PROMs), randomized controlled trials, Bone mineral density (BMD), radiostereometric analysis (RSA), femoral head, dual-energy X-ray absorptiometry, clinical outcomes, aseptic loosening

## Abstract

**Aims:**

Although the Fitmore Hip Stem has been on the market for almost 15 years, it is still not well documented in randomized controlled trials. This study compares the Fitmore stem with the CementLeSs (CLS) in several different clinical and radiological aspects. The hypothesis is that there will be no difference in outcome between stems.

**Methods:**

In total, 44 patients with bilateral hip osteoarthritis were recruited from the outpatient clinic at a single tertiary orthopaedic centre. The patients were operated with bilateral one-stage total hip arthroplasty. The most painful hip was randomized to either Fitmore or CLS femoral component; the second hip was operated with the femoral component not used on the first side. Patients were evaluated at three and six months and at one, two, and five years postoperatively with patient-reported outcome measures, radiostereometric analysis, dual-energy X-ray absorptiometry, and conventional radiography. A total of 39 patients attended the follow-up visit at two years (primary outcome) and 35 patients at five years. The primary outcome was which hip the patient considered to have the best function at two years.

**Results:**

At two and five years, more patients considered the hip with the CLS femoral component as superior but without a statistically significant difference. There were no differences in clinical outcome, magnitude of femoral component migration, or change of bone mineral density at five years. At three months, the Fitmore femoral component had subsided a median -0.71 mm (interquartile range (IQR) -1.67 to -0.20) and the CLS femoral component -0.70 mm (IQR -1.53 to -0.17; p = 0.742). In both groups the femoral head centre had migrated posteriorly (Fitmore -0.17 mm (IQR -0.98 to -0.04) and CLS -0.23 mm (IQR -0.87 to 0.07; p = 0.936)). After three months neither of the femoral components showed much further migration. During the first postoperative year, one Fitmore femoral component was revised due to aseptic loosening.

**Conclusion:**

Up to five years, we found no statistically significant difference in outcomes between the Fitmore and the CLS femoral components. The slightly worse outcomes, including one revised hip because of loosening, speaks against the hypothesis that the Fitmore femoral component should be advantageous compared to the CLS if more patients had been recruited to this study.

Cite this article: *Bone Jt Open* 2023;4(5):306–314.

## Introduction

Despite excellent survival rates of many contemporary femoral components, new designs, such as the Mayo stem (Zimmer Biomet, USA), have continuously been introduced often without adequate evaluation before market introduction.^1^ The stepwise algorithm proposed by Malchau^2^ has not always been followed.^3^ During the last decade, new legislation on medical devices has been adopted in the European Union.^4^ This legislation will, when fully implemented, put a greater responsibility on manufacturers to prove the safety and effectiveness of new implants.

Short femoral components were introduced in the 1980s to save proximal bone stock and facilitate later revision. However, there is little evidence that short femoral components perform better than implants of standard length, nor are their bone-sparing properties well established.^5,6^

The Fitmore Hip Stem (Zimmer Biomet) is defined as a short femoral component with mainly metaphyseal fixation. Although Fitmore has been on the European market since 2008, it has not been well documented in studies. There is, to the best of our knowledge, only one randomized controlled trial that has compared Fitmore to a femoral component of standard length with focus on bone remodelling.^7,8^ Other studies have suggested that Fitmore performs as well as contemporary femoral components, with good clinical outcomes and similar revision rates.^9-11^

In the present study, patients were operated with one-stage bilateral total hip arthroplasty using the Fitmore and the CementLeSs (CLS) Spotorno (Zimmer Biomet) femoral components on either side. Patients were followed for five years and clinical and radiological outcomes were recorded. The primary outcome was which of the two hips the patient regarded as best at two-year follow-up. Secondary outcomes were additional patient-reported outcome measures (PROMs), migration of the femoral component measured with radiostereometric analysis (RSA), change of bone mineral density, and revision for noninfectious reasons. We hypothesized that there would be no difference between the groups.

## Methods

### Study design

A total of 44 patients with bilateral osteoarthritis of the hip were recruited from the outpatient clinic at a single tertiary orthopaedic centre between 2011 and 2016 (Table I). Surgery was performed through one-stage bilateral total hip arthroplasty. The use of bilateral observations offers a unique opportunity to assess two different implants in one patient. This study design should minimize bias caused by individual differences in perception of pain and function, bone quality, bone turnover, and patient activity, all of which can influence femoral component fixation and changes in bone mineral density during the postoperative years.

**Table I.** Baseline demographic details.

Variable	Value
Patients, n	44
Mean age at surgery, yrs (range)	59 (43 to 73)
**Sex, n (%)**	
Male	22 (50)
Female	22 (50)
**Diagnosis n (%)**	
Primary osteoarthritis	39 (88)
Secondary osteoarthritis	2 (*5*)
Missing	3 (*7*)
Mean BMI, kg/m^2^ (range)	26.8 (19.7 to 35.2)
**ASA grade, n (%)**	
I	12 (27)
II	26 (59)
III	3 (7)
IV+	0
Missing	3 (7)
**Charnley classification, n (%)**	
A	0
B	15 (34)
C	14 (32)
Missing	15 (34)

ASA, American Society of Anesthesiologists; CI, confidence interval.

Inclusion criteria were hip anatomy suitable for both implants, bilateral end-stage osteoarthritis, general health compatible with bilateral one-stage surgery, and age between 35 and 70 years. Exclusion criteria were inability to read or understand the Swedish language, treatment with corticosteroids, osteopenia or osteoporosis, low lifetime expectancy, and ongoing oncological treatment. A research nurse conducted the randomization process using concealed envelopes. The most painful hip was randomized to either receive a Fitmore or a CLS femoral component, and the other hip was operated with the femoral component not used on the first side.

**Fig. 1:**
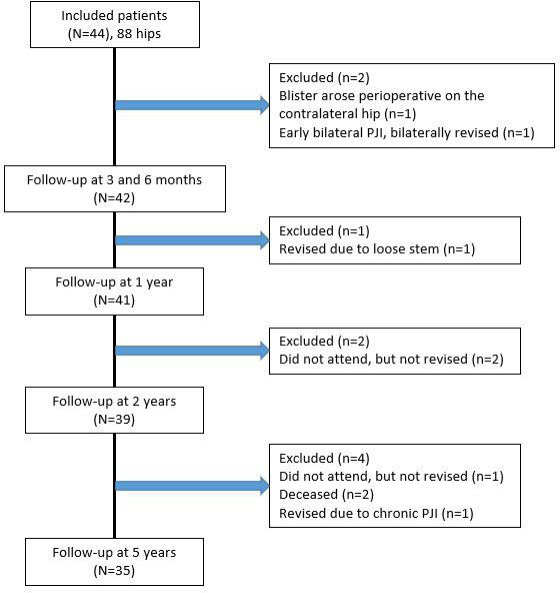
Flowchart of the 44 patients included in the study and the 35 remaining patients after five years. PJI, periprosthetic joint infection.

All operations were done through a direct lateral approach by four surgeons (including JK). The Trilogy cup (Zimmer Biomet) was used in all hips. Full weightbearing was allowed the day after the operation. The protocol was breached in one case, where a patient developed blisters on the contralateral hip during the operation of the first hip (CLS). Because of the risk of infection, the second hip was postponed; the patient remained in the study, but the results have not been included in the analyses. At two years, 39 patients remained in the study, and at five years 35 patients remained. (Figure 1).

### Fitmore

The Fitmore Hip Stem is a curved uncemented femoral component with a trapezoidal cross-section made of a titanium alloy (Ti-6Al-4V, Protasul-64WF). It is plasma spray coated with titanium alloy proximally and grit-blasted distally. The aim of the design is to achieve proximal metaphyseal fixation. The Fitmore system consists of three femoral component families (A, B, and C, with two offsets in family B), designed to cover different morphologies and to restore anatomy. The femoral component is categorized as a short implant.

### CLS Spotorno

The CLS Spotorno femoral component was first introduced in 1984, but the design was slightly changed in the 1990s. The femoral component has shown excellent results in national arthroplasty registries.^12,13^ It is a straight uncemented femoral component with a 3D taper, which aims to promotes stability even if the femoral component settles. In the sagittal plane, the femoral component has a trapezoidal cross-section. Proximal grooves enhance the area for bone ingrowth. The femoral component is made of a titanium alloy (Protasul-100) and has a grit-blasted surface. Three different caput-collum-diaphyseal angles (125°, 135°, and 145°) are available, but only 125° and 135° femoral components were used in this study. The CLS served as the reference.

### PROMs

Patients filled out a form (“My Hip”) containing four questions about preferred or best side with regard to limb strength, presence of lateral or thigh pain, instability, and overall function preoperatively and at two and five years. The patient could choose between neither, right, left, or both hips. “My Hip” is not a validated standard PROM. Further PROMs 36-item Short Form Survey (SF-36),^14^ Euro-Qol visual analogue scale (EQ-VAS),^15^ Euro-Qol five-dimension health questionnaire (EQ-5D),^16^ University of California, Los Angeles activity scale^17^ (UCLA), and VAS for satisfaction and pain were collected preoperatively and at three months and one, two, and five years postoperatively. VAS for pain and satisfaction were rated 0 to 10, where 0 is best possible outcome and 10 is the worst possible outcome. The forms were sent out by mail and then collected at the visit to the outpatient clinic. VAS-pain and VAS-satisfaction were filled out separately for each hip during follow-up. Preoperatively, the overall pain was indicated without any separation between the two sides. Likewise, separate evaluation of the two hips was accidently missed in ten cases at five years. Therefore, only 25 patients with bilateral forms for VAS pain and VA satisfaction are accounted for. The Harris Hip Score (HHS)^18^ was filled out bilaterally by the physician preoperatively and at one, two, and five years postoperatively.

## RSA

During surgery, between seven and nine 0.8 mm tantalum markers were placed in the proximal femur bone. Translation of the femoral head centre represented migration of the femoral component. Plain radiographs were taken with two detectors at 40° between tubes. A uniplanar cage was used (Cage 77; RSA Biomedical, Sweden). Examinations were conducted postoperatively (median 3.5 days (interquartile range (IQR) 2 to 5), and at three and six months and one, two, and five years after the operation. Double examinations were done in 85 hips postoperatively. The smallest detectable motions in an individual case at the 99 percent significance limit were 0.28 mm, 0.22 mm, and 0.70 mm for medial (+) or lateral (-), proximal (+) or distal (-), and anterior (+) or posterior (-) translations, respectively, corresponding to the standard deviation of the error multiplied by 2.66 and based on a supposed zero-value of the mean difference between the two examinations. At five years, the median value of mean error of rigid body fitting was 0.18 (IQR 0.15 to 0.22), the median condition number 31 (IQR 26 to 42), and the median number of used markers in the femoral reference segment was 7 (IQR 5 to 8). In one patient, RSA examinations exceeded the RSA guidelines for condition number (> 150). This patient’s RSA examinations for both hips were excluded. One patient did not attend the five-year follow-up but was not revised. Another patient had missing data at six months; these results were extrapolated. All other patients (n = 32) had complete RSA follow-up at five years. The analyses include all cases up to any exclusion regardless of reason.

### Bone mineral density and radiological evaluation

Dual-energy X-ray absorptiometry (DXA) scans were performed with a Hologic Discovery QDR DXA scanner and Hologic Discovery Apex software v. 12.7.3 (Hologic, USA). The metal removal program was used as scan mode. Changes in bone mineral density between the postoperative examination and at three and six months and one, two, and five years were related to the seven Gruen zones.^19^

Conventional radiological examinations (anteroposterior (AP), lateral, and pelvic view) were carried out postoperatively at three months and one, two, and five years. The radiographs were analyzed for radiolucent lines on the AP and lateral view. Parts of the results up to two years with focus on gait analysis (22 patients) have been previously published.^20^ One author (KR) analyzed radiographs at five years using MDesk software (v. 4.0.7; RSA Biomedical).

### Statistical analysis

The results from the “My Hip” questionnaire including preferred or best hip were cross tabulated and analyzed using Fisher’s exact test. All other PROM and RSA data were non-normally distributed and Wilcoxon signed-rank test was used for evaluation. Data distribution was determined using plotting and tests of normality (Kolmogorov-Smirnov, Shapiro-Wilk).

Bone mineral density (BMD) loss was normally or close to normally distributed, hence paired *t*-test was used. All tests were two-sided and the significance level was set to < 5%, except for calculation of the precision of the RSA measurements, where it was set to < 1%. SPSS v. 24.0.00 (IBM, USA) was used for all analyses.

We assumed that there would be 35 remaining observations at two years. Provided that at least 26 of them preferred one of femoral components, a study power exceeding 80 percent should be reached.

## Results

### PROM

Our primary PROM was which of the hips that the patient considered to be best at two years. Although more patients considered the hip operated with CLS to be better than the Fitmore hip at both two and five years, the difference was not statistically significant (p = 0.289 and p = 0.240, respectively; Fisher’s exact test) (Table II). The same pattern applied to all questions in the “My Hip” questionnaire. Patients tended to be more satisfied with the CLS hip but without statistical significance. Clinical outcome improved during the first 12 months, but thereafter no further improvement was seen. No statistical difference could be seen in any of the additional PROM or HHS data (Table III).

**Table II.** Distribution of answers in the My Hip form.

Question	Preoperative	Two years	p-value*†	Five years	p-value*‡
**Which hip is strongest?**	41	37	0.131	33	0.789
Similar	16	14		13	
Fitmore	15	8		9	
CLS	10	15		11	
**Do you have pain in or on the outside of your thigh?**	41	37	0.787	33	0.764
Both	36	3		2	
Fitmore	1	6		6	
CLS	3	3		4	
Neither	1	25		21	
**Do you consider your hip to be unstable?**	41	37	0.358	34	0.613
Both	13	0		0	
Fitmore	0	4		3	
CLS	5	1		1	
Neither	23	32		29	
**Which hip has the best overall function?**	41	37	0.287	34	0.240
Both	15	28		18	
Fitmore	14	7		5	
CLS	12	12		10	

* Fisher’s exact test.

† Preoperative vs two years.

‡ Two years vs five years.

**Table III.** Patient-reported outcome measures preoperatively and at one, two, and five years. General health instruments are registered once for each patient.

Instrument	Preoperative		3 mths		1 yr		2 yrs		5 yrs		p-value*	
	n	Median (IQR)	n	Median (IQR)	n	Median (IQR)	n	Median (IQR)	n	Median (IQR)	2 yrs	5 yrs
**General health**												
SF-36 Mental	37	47 (38 to 54)	0		39	55 (47 to 58)	39	55 (50 to 58)	33	55 (48 to 57)	N/A	N/A
SF-36 Physical	37	23 (18 to 29)	0		39	48 (38 to 56)	39	51 (40 to 55)	33	50 (41 to 54)	N/A	N/A
EQ-5D	40	0.68 (0.59 to 0.76)	42	0.80 (0.71 to 0.91)	38	0.95 (0.87 to 0.97)	39	0.97 (0.87 to 0.97)	33	0.93 (0.87 to 0.97)	N/A	N/A
EQ-VAS	40	38 (30 to 64)	42	75 (65 to 90)	38	85 (75 to 95)	38	90 (75 to 95)	32	81 (75 to 90)	N/A	N/A
UCLA	39	3 (3 to 6)	39	5 (4 to 6)	37	6 (6 to 7)	36	6 (6 to 8)	32	6 (6 to 7.5)	N/A	N/A
**Hip-specific**												
**VAS pain†**											0.651	0.285
Fitmore	40	70 (60 to 77)	40	20 (4 to 36)	35	3 (0 to 15)	37	2 (0 to 13)	25	5 (0 to 27)		
CLS	40	70 (60 to 77)	40	10 (2 to 30)	35	1 (0 to 5)	37	2 (0 to 14)	25	5 (0 to 17)		
**VAS satisfaction**											0.585	0.310
Fitmore	40	N/A	40	10 (1 to 29)	35	2 (0 to 16)	37	3 (0 to 16)	25	3 (0 to 25)		
CLS	40	N/A	40	5 (0 to 23)	35	1 (0 to 9)	37	2 (0 to 14)	25	3 (0 to 23)		
**Harris Hip Score**											0.469	0.721
Fitmore	21	43 (43 to 56)	0	N/A	39	98 (95 to 100)	37	99 (96 to 100)	33	99 (99 to 100)		
CLS	21	42 (31 to 53)	0	N/A	39	98 (95 to 99)	37	99 (96 to 100)	33	99 (97 to 100)		

* Wilcoxon signed-rank test.

† Assessed bilaterally.

EQ-5D, EuroQol five-dimension health questionnaire; IQR, interquartile range; N/A, not applicable; SF-36, 36-Item Short Form survey; UCLA, University of California, Los Angeles Activity Scale; VAS, visual analogue scale.

### RSA

**Fig. 2:**
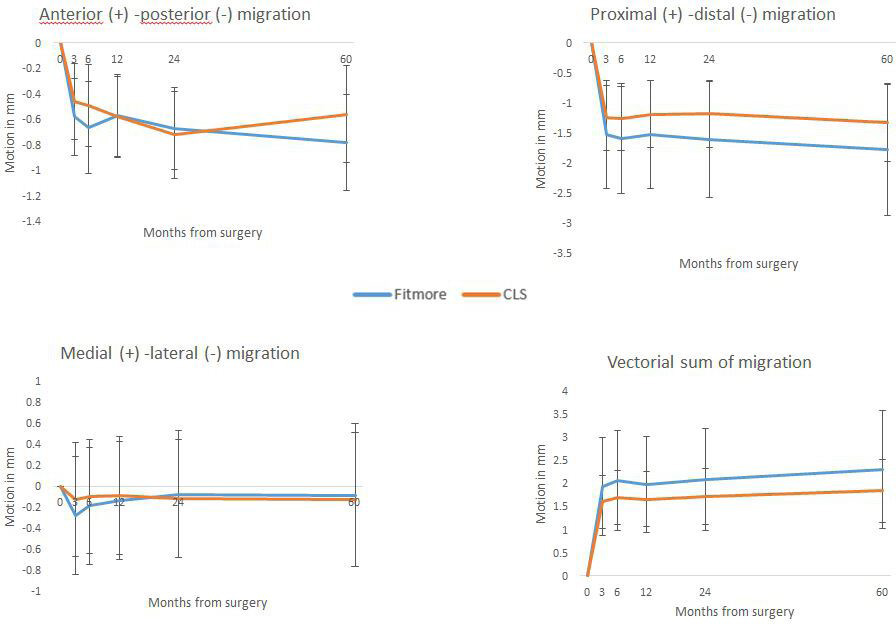
Mean migration in mm ± 2 standard errors of the mean.

Up to three months, both components showed a distal migration (Fitmore, median -0.71 mm (IQR -1.67 to -0.20); CLS, median -0.70 mm (IQR -1.53 to -0.17); p = 0.742, Wilcoxon signed-rank test) and posterior migration (Fitmore, median -0.17 mm (IQR -0.98 to -0.04); CLS, median -0.23 mm (IQR -0.87 to 0.07); p = 0.936, Wilcoxon signed-rank test). The median migration in the medial-lateral directions was 0.15 mm (IQR -0.39 to 0.36) on the Fitmore side and -0.04 mm (IQR -0.25 to 0.15), on the control side (p = 0.320, Wilcoxon signed-rank test; Figure 2). After three months the components had stabilized with little further migration (Table IV).

**Table IV.** Migration of the femoral head centre between examinations.

Component type	Median, mm (IQR)					p-value†	
	0 to 3 mths	3 to 6 mths	6 to 12 mths	1 to 2 yrs	2 to 5 yrs	0 to 3 mths	2 to 5 yrs
Patients, n	40	39	38	38	32		
**Medial (+) - lateral (-)**							
Fitmore	0.15 (-0.39 to 0.36)	0.06 (-0.05 to 0.14)	0.04 (-0.07 to 0.12)	0.05 (-0.07 to 0.15)	0.07 (-0.04 to 0.13)	0.320	0.675
CLS	-0.04 (-0.25 to 0.15)	-0.01 (-0.08 to 0.06)	-0.004 (-0.09 to 0.08)	0.03 (-0.06 to 0.07)	0.03 (-0.05 to 0.08)		
**Proximal (+) - distal (-)**							
Fitmore	-0.71 (-1.67 to -0.20)	-0.01 (-0.06 to 0.07)	-0.04 (-0.13 to 0.06)	0.02 (-0.07 to 0.06)	-0.01 (-0.15 to 0.07)	0.742	0.694
CLS	-0.70 (-1.53 to -0.17)	-0.02 (-0.07 to 0.05)	0.02 (-0.05 to 0.07)	0.02 (-0.05 to 0.07)	-0.02 (-0.09 to 0.06)		
**Anterior (+) - posterior (-)**							
Fitmore	-0.17 (-0.98 to -0.04)	-0.06 (-0.32 to 0.14)	-0.04 (-0.17 to 0.14)	-0.05 (-0.17 to 0.12)	-0.07 (-0.21 to 0.13)	0.936	0.177
CLS	-0.23 (-0.87 to 0.07)	-0.01 (-0.31 to 0.11)	-0.05 (-0.25 to 0.10)	-0.06 (-0.19 to 0.05)	-0.01 (-0.11 to 0.08)		
Total*							
Fitmore	0.87 (0.32 to 1.94)	0.25 (0.14 to 0.44)	0.22 (0.16 to 0.37)	0.22 (0.15 to 0.37)	0.23 (0.17 to 0.35)	0.946	0.058
CLS	1.04 (0.30 to 1.92)	0.22 (0.16 to 0.36)	0.21 (0.16 to 0.34)	0.21 (0.13 to 0.34)	0.15 (0.12 to 0.25)		

* Vectorial sum of migration.

† Wilcoxon signed-rank test.

IQR, interquartile range.

Between two and five years, one Fitmore showed migration above the detection limit in both the proximal-distal and anterior-posterior directions. Another six stems (three Fitmore and three CLS) migrated above the detection limit only in the proximal-distal direction. No components showed any movement above the detection limit in the medial-lateral direction. The patient with migration in two directions reported excellent clinical results and no other radiological signs of loosening could be detected.

### DXA

**Fig. 3:**
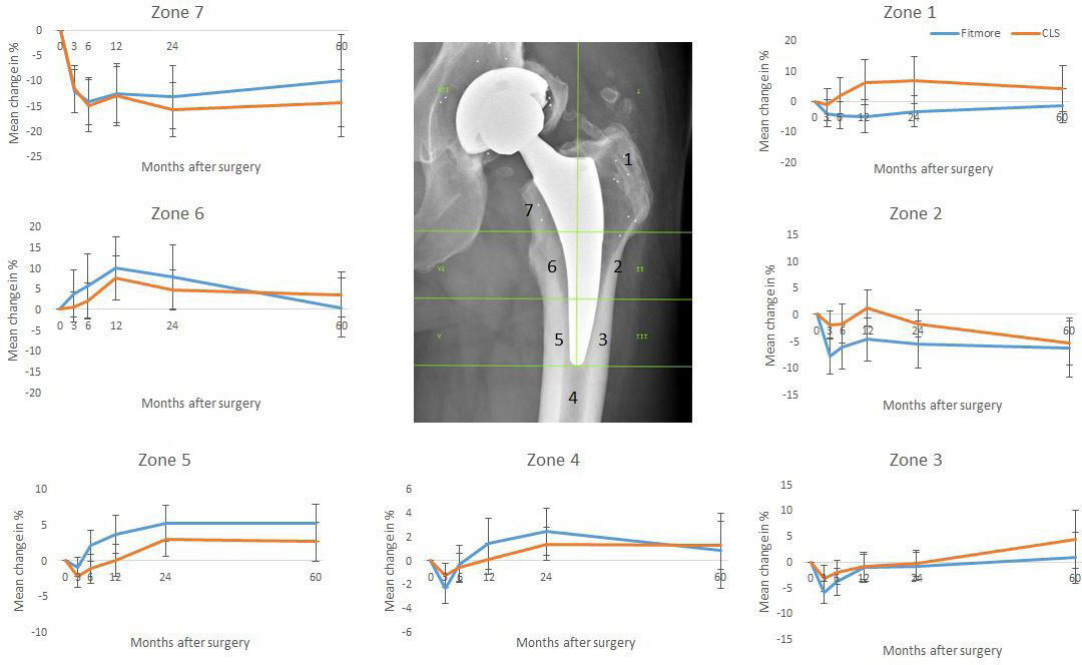
Change in bone mineral density loss in % related to Gruen zones. Mean ± 2 standard errors of the mean.

Only patients with examinations at all occasions were included in the DXA analysis (n = 27). Both components showed similar patterns of BMD loss (Figure 3). Great loss of BMD was seen in Gruen zone seven: this occurred during the first postoperative year and was then stable. No statistical difference could be seen between groups in zone seven at either at two or five years (p = 0.444 and p = 0.391, paired *t*-test). In Gruen zone one, the Fitmore lost slightly more BMD. At two years the difference was statistically significant (p = 0.001, paired *t*-test), but became statistically insignificant at five years (p = 0.088, paired *t*-test).

### Radiological evaluation

At five years, a total of nine hips (six Fitmore and three CLS) showed radiolucent lines in any Gruen zone. In two cases (one Fitmore and one CLS), there were radiolucencies on both the frontal and the lateral view. Another five Fitmore and two CLS components showed radiolucencies only on the lateral or the frontal view. No stem had radiolucencies exceeding 20% of the total implant to bone interface in any of the views. The median percentage of radiolucencies was 0% (IQR 0% to 0%) in both groups.

### Revisions

Overall, three patients (four hips) were revised. One patient had an early bilateral periprosthethic infection and was bilaterally revised. One hip (Fitmore) was revised at approximately ten months postoperatively due to aseptic loosening. Before the two-year follow-up, one hip (Fitmore) was diagnosed with chronic infection and was revised. In the cases with uni- or bilateral revision, data from both hips were excluded from the date of revision.

## Discussion

In this randomized controlled trial, we found no significant difference between the Fitmore and CLS components. More patients tended to regard the CLS as the better hip at two years, but the difference was not statistically significant. Moreover, both showed an initial migration followed by stabilization. At five years proximal BMD loss did not differ between the groups. One Fitmore had been revised due to noninfectious loosening.

Since the patients in our study were operated bilaterally, the status of both hips will be reflected in the general PROM data. Nevertheless, patients report improvement of their general physical and mental health. Our findings are supported by previous studies.^8-11^ In the hip-specific evaluations, no difference could be seen between the groups. The median HHS almost reached the highest possible value, a reminder of the ceiling effects with this instrument.^21^ The Forgotten Joint Score^22^ might have a more advantageous profile considering ceiling effects, but was not available to us when this study was initiated.

Femoral components in both groups showed an early migration followed by stability. Earlier studies have shown similar patterns with both the Fitmore and CLS.^9,23^ With cemented composite beam femoral components, early migration predicts loosening,^24,25^ but this does not seem to be the case with uncemented femoral components, regardless of length. Evidence shows that early settling followed by stabilization does not threaten the implant survival in the long run.^26-29^

BMD loss around implants (stress shielding) is a phenomenon known to occur with all implants due to altered load transfer. One previous study has shown results comparable to ours.^8^ Proximal bone resorption and, with time, increased distal BMD, suggest diaphyseal fixation and unloading of the proximal femur. In our study this was reflected by a slight increase in zones four and five at five years in both groups, and also in zones three and six in the CLS group. Additionally, the CLS group showed an increase in zone five, maybe because of its tapered shape. Pepke et al^30^ conducted a biomechanical study comparing the CLS with the Fitmore femoral component in synthetic bone models, showing that Fitmore is slightly stiffer in the mediolateral plane, which entails a greater load distally even though the CLS is longer. Proximal BMD loss is not believed to cause aseptic loosening, but might complicate future revision surgery.^31^

Short femoral components have risen in popularity despite little evidence for their superiority over those of standard length.^5,6^ The rationale for using short femoral components is that they should be compatible with improved hip biomechanics, preserve proximal femoral anatomy, and thereby also facilitate any future revision. There are, to our knowledge, no studies on revision of short femoral components. Although this theoretical advantage is a widespread belief, it must be studied further. In the few studies of gait analysis performed on short femoral components, no difference could be detected when compared to femoral components of standard length.^20,32,33^ In addition, the degree of return to sports does not seem to be affected by component length, but rather by the degree of physical activity before surgery.^34,35^

The strength of this study is that the femoral components are evaluated with a broad spectrum of clinical and radiological tools. All patients received the same acetabular implant and were operated through the same incision, which increases the probability that any differences depend on the choice of femoral component.

Since patients were operated bilaterally and therefore served as their own control, the risk of bias caused by individual differences in bone quality is minimized. On the other hand, our study has limitations such as the lack of high-precision clinical evaluation tools, and the fact that our primary PROM is not a validated tool. There is, to the best of our knowledge, no validated PROM for bilateral assessment. Lack of statistically significant differences for the other validated PROMs filled in bilaterally by the patients are, however, in line with the results of our primary outcome. Furthermore, migration was measured as translation of the femoral head, and we could not record femoral component rotations. Model-based RSA including the Elementary Geometrical Shapes application was not available to us when this study was started, and the distal part of the femoral component was not visualized on the RSA radiographs. However, sufficiently relevant information can be obtained even if recordings are limited to femoral head migration.^25,36,37^

In our study no advantages could be found to promote a widespread use of the Fitmore. The slightly worse outcomes, including one revised hip because of loosening, speaks against the hypothesis that the Fitmore should be advantageous compared to the CLS if more patients had been recruited to this study. Further multicentre RCTs are needed, preferably using national registries, to reject or confirm our observations.

**Take home message**

- The Fitmore femoral component does not have a better clinical outcome than the CLS femoral component.

- No radiological benefits or bone-sparing properties could be seen with the Fitmore femoral component.
